# Nanoparticle-Mediated Radiotherapy: Unraveling Dose Enhancement and Apoptotic Responses in Cancer and Normal Cell Lines

**DOI:** 10.3390/biom13121720

**Published:** 2023-11-29

**Authors:** Maria Anthi Kouri, Ellas Spyratou, Maria-Eleni Kalkou, Georgios Patatoukas, Evangelia Angelopoulou, Ioanna Tremi, Sophia Havaki, Vassilis G. Gorgoulis, Vassilis Kouloulias, Kalliopi Platoni, Efstathios P. Efstathopoulos

**Affiliations:** 1 2nd Department of Radiology, Medical School, Attikon University Hospital, National and Kapodistrian University of Athens, 11527 Athens, Greece; mariakouri90@gmail.com (M.A.K.); spyratouellas@gmail.com (E.S.); gpatatouk@gmail.com (G.P.); vkouloul@med.uoa.gr (V.K.); polaplatoni@gmail.com (K.P.); 2 Medical Physics Program, Department of Physics and Applied Physics, Kennedy College of Sciences, University of Massachusetts Lowell, 265 Riverside St., Lowell, MA 01854, USA; 3 Physics Department, School of Applied Mathematical and Physical Sciences, National Technical University of Athens, Iroon Polytechniou 9, 15780 Athens, Greece; 4 Medical School, National and Kapodistrian University of Athens, 75 Mikras Asias Str., 11527 Athens, Greece; marilou94kal@gmail.com; 5 2nd Department of Pathology, School of Medicine, Attikon University Hospital, National and Kapodistrian University of Athens, 12462 Athens, Greece; eva.angelop@gmail.com; 6 Molecular Carcinogenesis Group, Department of Histology and Embryology, Medical School, National and Kapodistrian University of Athens, 11527 Athens, Greece; ioannatre@med.uoa.gr (I.T.); shavaki@med.uoa.gr (S.H.); vgorg@med.uoa.gr (V.G.G.); 7 Biomedical Research Foundation, Academy of Athens, 11527 Athens, Greece; 8 Ninewells Hospital and Medical School, University of Dundee, Dundee DD1 9SY, UK; 9 Faculty Institute for Cancer Sciences, Manchester Academic Health Sciences Centre, University of Manchester, Manchester M20 4GJ, UK; 10 Faculty of Health and Medical Sciences, University of Surrey, Surrey GU2 7YH, UK

**Keywords:** gold nanoparticles, radiation therapy, SiHa, Caski, HCK1T, cellular apoptosis, dose enhancement, linear quadratic model

## Abstract

Cervical cancer remains a pressing global health concern, necessitating advanced therapeutic strategies. Radiotherapy, a fundamental treatment modality, has faced challenges such as targeted dose deposition and radiation exposure to healthy tissues, limiting optimal outcomes. To address these hurdles, nanomaterials, specifically gold nanoparticles (AuNPs), have emerged as a promising avenue. This study delves into the realm of cervical cancer radiotherapy through the meticulous exploration of AuNPs’ impact. Utilizing ex vivo experiments involving cell lines, this research dissected intricate radiobiological interactions. Detailed scrutiny of cell survival curves, dose enhancement factors (DEFs), and apoptosis in both cancer and normal cervical cells revealed profound insights. The outcomes showcased the substantial enhancement of radiation responses in cancer cells following AuNP treatment, resulting in heightened cell death and apoptotic levels. Significantly, the most pronounced effects were observed 24 h post-irradiation, emphasizing the pivotal role of timing in AuNPs’ efficacy. Importantly, AuNPs exhibited targeted precision, selectively impacting cancer cells while preserving normal cells. This study illuminates the potential of AuNPs as potent radiosensitizers in cervical cancer therapy, offering a tailored and efficient approach. Through meticulous ex vivo experimentation, this research expands our comprehension of the complex dynamics between AuNPs and cells, laying the foundation for their optimized clinical utilization.

## 1. Introduction

Cervical cancer represents a significant health threat as in 2020, an estimated 604,000 women were diagnosed globally [1]. This type of cancer presents a challenge in its proclivity to remain undetected until its advanced stages. Therefore, the absence of discernible symptoms in the early phases underscores the significance of an effective, targeted therapeutic method [2].

Radiotherapy, a cornerstone in cervical cancer treatment, utilizes ionizing radiation to induce targeted cellular damage. Despite its efficacy, challenges like targeted dose deposition and radiation exposure to healthy tissues persist, hindering optimal outcomes. Innovative strategies, including the integration of nanomaterials like gold nanoparticles (AuNPs), have gained traction. AuNPs, with their high atomic number (Z), demonstrate strong photoelectric absorption coefficients and thus robust photon attenuation, excellent biocompatibility, and relatively low biological toxicity [3]. Their physical, chemical, and biological mechanisms, that make them excellent candidates as emerging tumor radiosensitizers, have been analyzed by the scientific community during the past decades [4]. AuNPs present low permeability in normal tissues through the enhanced permeability and retention (EPR) effect and low systemic clearance [5,6,7]. However, the exact mechanism underlying the dose enhancement caused by gold nanoparticles have not been fully understood and explained as it constitutes a multifactorial problem [7].

Their irradiation with photons leads to a subsequent cascade of secondary interactions, low energy photoelectrons and Auger electrons [8]. Due to their very short range in biological matter (nm-μm)—a similar range to the width of the DNA strand—they manage to effectively cause direct damage to the cancerous DNA but also indirect damage and oxidative stress, since the radiation interacts with water molecules as well as with other organic molecules within the cell and eventually leads to the production of highly reactive free radicals [9]. Both the direct as well as the indirect action of the combined efforts of ionizing radiation and AuNPs leads to single-strand breaks (SSBs) and double-strand breaks (DSBs) of the DNA [10,11].

Tumor cells have the ability to efficiently repair radiation-induced damage, a fact that strongly influences their intrinsic radiosensitivity. If cancer cells manage to efficiently activate their repair mechanisms, they may induce programmed cell death or apoptosis to prevent the accumulation of mutations in daughter cells [12]. After exposure to ionizing radiation, cancer cells can undergo apoptosis as a protective mechanism. The apoptotic mechanisms can be related with DNA Damage Recognition, Mitochondrial Pathways, Death Receptors and Extrinsic Pathways, Inflammatory Response Suppression, or Cell Fragmentation and Phagocytosis [13,14].

Normal cells on the other hand, process robust DNA repair mechanisms to maintain their genomic stability. The efficiency and accuracy of DNA repair mechanisms can be influenced by the cell type as well as the post-irradiation time of each repair process [14,15,16,17]. Different cell types may have varying levels of repair enzymes and proteins, impacting their ability to repair DNA damage. Additionally, repair mechanisms can be influenced by the cell cycle phase; for example, cells in the S phase are actively replicating their DNA and may have more robust repair mechanisms compared to cells in other phases [18]. Moreover, the efficiency of repair mechanisms tends to decline with age, leading to an accumulation of DNA damage over time. These factors collectively determine how cells respond to DNA damage and their ability to maintain genomic stability.

Consequently, the enhancement of localized radiosensitization is a vital parameter to consider in the treatment of cervical cancer patients. It directly affects the resulting biological damage at the tumor site, the simultaneous protection and thus the recovery time of the surrounding healthy tissue, and eventually the total outcome of the patient’s treatment after radiation therapy.

The purpose of this study was to evaluate the biological dose response with and without AuNPs in cancer and normal cervical cell lines and to quantify the dose enhancement factor and the apoptotic programmed cell death for different deposited doses and post-irradiation times after using a 6 MV photon energy Medical Linear Accelerator. That level of energy, despite being way above the Photoelectric Range where AuNPs have been proven to be more effective, constitutes the current clinical standard serving as the reference radiation. Many studies have demonstrated that the use of AuNPs in radiotherapy at clinical MV energies can increase the deposit dose in the target volumes [19,20]. Dose enhancement ratios ranging from 14 to 287% were observed using gold nanoshells with 6 MV Linac beams [21].

Furthermore, the experimental research determines the ideal post-irradiation interval during which AuNPs induce maximal damage to cancer cells, preventing substantial repair, and also examining the same effects on normal cells. Moreover, this study examined the optimal application of AuNPs as radiosensitizers by observing apoptosis levels. This approach enabled the precise quantification of radiobiological interactions for cancer and normal cells at various post-irradiation intervals. A better understanding of the AuNPs interactions with biological matter within this range of these energies will have implications for future translational research.

## 2. Materials and Methods

### 2.1. Cell Culturing Protocol

Two cervical cancer cell lines and one normal cervical epithelium cell line were used. The human cervical cancer cell lines SiHa and CaSki were obtained from the ATCC (American Type Culture Collection). The cell lines were grown and maintained separately in the appropriate Dulbecco minimum essential media (DMEM) with 10% fetal bovine serum (FBS), 1% penicillin, and 1% amphotericin B according to the instructions supplied by the vendor. The cell line HCK1T (Human Cervical Keratinocytes), a normal cervical epithelium cell line, was kindly offered by Tohru Kiyono [22] and was cultured as proposed [23] using Defined Keratinocyte Serum-Free Medium (SFM) supplemented with 5 ng/mL Epidermal Growth Factor (EGF) and 50 μg/mL of Bovine Pituitary Extract (BPE). The cell lines cells were dispensed into separate 75 cm^2^ tissue culture flasks and were incubated at 37 °C, 5% CO_2_ air atmosphere. Before the treatment day, the cells, at a confluency of 70–80% in serum-supplemented media were trypsinized and harvested, and the pellets were washed in phosphate buffered saline (PBS) 3 times. They were plated on 24-well, flat bottom plates and half of the samples of each plate were incubated with the nanoparticles at a specific concentration (5 μg/1 mL media) for 24 h at 37 °C allowed to attach overnight using the DMEM medium with 10% FBS.

### 2.2. Gold Nanoparticles (AuNPs)

Polyethylene Glycol (PEG) coating gold nanoshells (AuNSs) purchased from NanoComposix (San Diego, CA, USA) have been used as radiosensitizers. The gold nanoshells were 120 nm in diameter and their gold core was surrounded by a gold shell 16 nm in thickness. PEG coating increases stability and biocompatibility and prevents particle aggregation [24]. The AuNPs were incubated with normal cervical and cervical cancer cell lines for 24 h.

### 2.3. AuNPs Cellular Uptake using Transmission Electron Microscopy (TEM)

For cellular uptake studies, SiHa cells were grown in 100 mm Petri dishes. After treatment with AuNPs (for 24 h), cells were washed thrice with PBS and fixed in 2.5% glutaraldehyde solution in 0.01 M PBS, pH 7.2–7.4, for 30 min. After fixation, cells were harvested using scraper, centrifuged at 800× *g* for 5 min at RT and finally embedded in 4% gelatin aqueous solution. The standard procedure for TEM processing of specimens (cells–gelatin fragments) was followed, i.e., post-fixation with OsO_4_, dehydration, infiltration, and embedding in epoxy resins. Epoxy blocks were then cut into thin sections (~80 nm thickness), which were mounted on copper grids, stained with alcoholic uranyl acetate and lead citrate, and finally observed and photographed using FEI Morgagni 268 TEM, operated at 80 kV accelerating voltage with an objective aperture of 30 μm and equipped with a digital CCD camera (Olympus, Morada, Tokyo, Japan).

### 2.4. LINAC Cell Irradiation

For irradiation experiments, 24-well cell culture plates were seeded with 200,000 cells/well in DMEM media supplemented with 10% FBS 24 h prior to treatment with and without AuNPs. Four sets of 24-well plates were prepared for each cell line (SiHa, Caski and HCK1T), in which one plate was irradiated to receive 1 Gy of deposited dose, one to receive 2 Gy, and one to receive 4 Gy, respectively, whereas the non-irradiated plate (0 Gy of deposited dose) served as control.

Irradiation conditions for all plates included the utilization of a Medical Linear Accelerator located in the Radiotherapy Unit (2100 CLINAC, Varian, Palo Alto, CA, USA) using a 6 MV photon energy and two anti-parallel fields; one from the anterior direction (AP, LINAC gantry angle = 0 degrees) and one from the posterior direction (PA, LINAC gantry angle = 180 degrees). Field size was defined by jaws and set at 15 × 15 cm at isocenter distance (Source Axis Distance, SAD = 100 cm) to ensure adequate geometric and dosimetric coverage of the plate. Source surface distance (SSD) was 91.6 and 95.6 for the AP and PA fields, respectively. Dose rate was set at 240 Monitor Units (MU) per minute.

**Figure 1:**
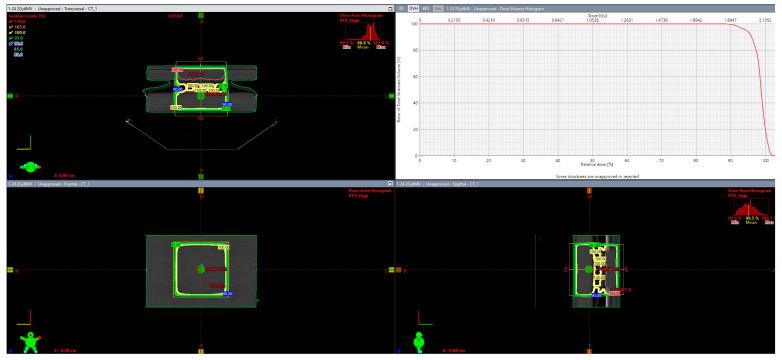
Dose delivery environment on eclipse treatment planning (version 17, Varian, Palo Alto) depicting the 24−well plates with cells and cells with AuNPs receiving 2 Gy at 6 MV. The transversal, frontal, and sagittal planes are illustrated as well as the Dose Volume Histogram.

Furthermore, to ensure adequate dose coverage both in the entrance and the exit regions of each plate, water equivalent bolus material (size 30 × 30 cm) as well as PMMA slabs (30 × 30 cm, density 1.19 g/cm^3^) were used in such a way that all plates were sandwiched between 0.5 cm bolus and 5 cm PMMA slabs in the PA direction and 1 cm bolus and 4 cm PMMA slabs in the AP direction. Dose delivery was calculated using the eclipse treatment planning (version 17, Varian, Palo Alto) and the Analytic Anisotropic Algorithm (AAA) as can be depicted in Figure 1.

The resulting MU were the following: 1:59 (AP) and 55 (PA) for the 1 Gy irradiation scheme, 118 (AP) and 110 (PA) for the 2 Gy irradiation scheme, and 237 (AP) and 219 (PA) for the 4 Gy irradiation scheme. Confirmation of the dose coverage was assessed using both dose distribution and dose statistics in terms of dose volume histogram DVH (see images). In all three cases, at least 99% of the target (plate) received 95% of the prescribed dose as per ICRU guidelines.

### 2.5. Cell Viability and Apoptosis Assay

**Figure 2:**
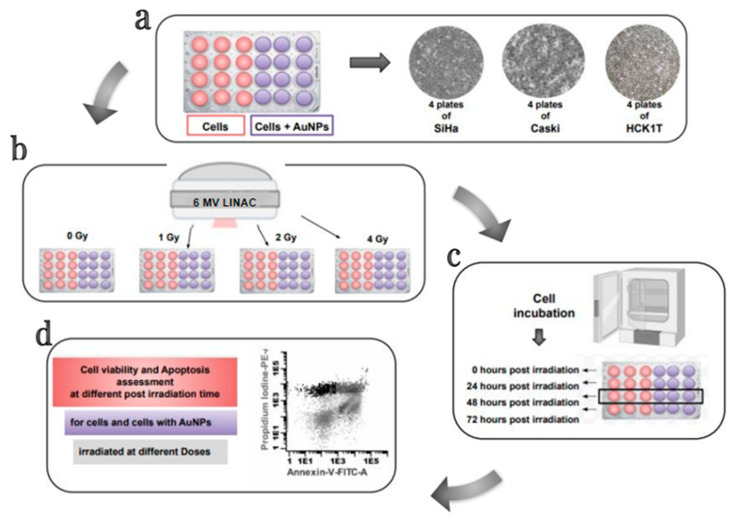
Schematic Illustration of the experimental set up and methodology: (**a**) 24-well cell culture plates arrangement (cells and cells with AuNPs) for the two cervical cancer cell lines and the normal cervical cell line. (**b**) Irradiation conditions and deposited doses for each cell plate (cells and cells with AuNPs) at the Medical Linear Accelerator. (**c**) Post-irradiation incubation of cell plates (cells and cells with AuNPs for 0 h, 24 h, 48 h, and 72 h. (**d**) Annexin labelling, apoptosis and cell viability assessment with flow cytometry.

Cell viability was assessed with flow cytometry on an Omnicyt (Cytognos, Salamanca Spain). The irradiated four different doses (0 Gy, 1 Gy, 2 Gy, 4 Gy), cell suspensions with and without 120 nm AuNPs, were labelled with Annexin V (Alexa Fluor 488, Dead cell Apoptosis Kit, Invitrogen, Thessaloniki, Greece). After irradiation, cells were incubated for 0 h, 24 h, 48 h, and 72 h post irradiation. Each group was stained and counted by the flow cytometer. The entirety of the experimental methodology can be depicted in Figure 2.

### 2.6. Clonogenic Survival Assay and Data Processing

Clonogenic cell survival assay was performed to evaluate the therapeutic effect of radiation on the survival of cancer and normal cervical cells with and without AuNPs for different doses produced by a 6 MeV clinical LINAC. The relative cell surviving fraction was calculated with the aid of the following Equations (1) and (2):

(1) $$Platting\;Efficiency\;\left(PE\right)\left(\%\right)=\frac{No.\;of\;Colonies\;formed\;}{No.\;of\;cells\;seeded}\times 100$$

(2) $$Surviving\;Fraction\;\left(SF\right)\left(\%\right)=\frac{No.\;of\;Colonies\;formed\;after\;treatment\;}{No.\;of\;cells\;seeded\times PE}\times 100\;$$

What those equations describe is that the *Platting efficiency (PE)* characterizes the ratio of the number of colonies to the number of the initial cells seeded. After treatment, the number of colonies counted is expressed in terms of *PE* but now defined as the *Survival Fraction (SF).*

To further predict the impact of radiotherapy treatment with and without AuNPs and correlate it with the physics of radiation interactions in different deposited doses as well as with the radiobiological effects and the cell responses after radiation damage to the DNA, we used the linear quadratic (LQ) model. The equation that describes this key tool in preclinical radiobiological modeling of cell survival as a function of dose is Equation (3):

(3) $$SF=e^{-\alpha D-\beta D^{2}}$$

where *SF* is the survival fraction as described by Equation (2), *D* is the deposited dose. *α* parameter represents the linear component of the equation, indicating the sensitivity of cells to low doses of radiation. *β* represents the quadratic component, indicating the sensitivity of cells to higher doses of radiation.

A MATLAB-based code (MATLAB R2022b) was developed to estimate *α/β* parameters and to visualize the *surviving fraction* (*SF*) curves over the dose for different post-irradiation times. The same code was used to calculate the *Dose Enhancement Factor* (*DEF*), meaning the radiosensitization that is microscopically achieved with the presence of AuNPs over the absorbed dose without AuNPs, as described in Equation (4):

(4) $$DEF=\frac{D_{0,NP}}{D_{0,cont}}\;$$

MATLAB code was also developed to visualize the apoptotic responses of cells with and without AuNPs in correlation with the 4 different post-irradiation times for the different doses.

### 2.7. Statistical Analysis

Statistical comparisons for the DEF values among the different irradiation conditions (*n* = 4 biological replicates) were performed using the non-parametric Wilcoxon–Mann–Whitney test. Different correlations were tested, comparing the cell lines, the doses, and the post-irradiation time. Differences were considered significant at *p* value < 0.05.

## 3. Results

### 3.1. Distribution and Localization of AuNPs in Cells via TEM

**Figure 3:**
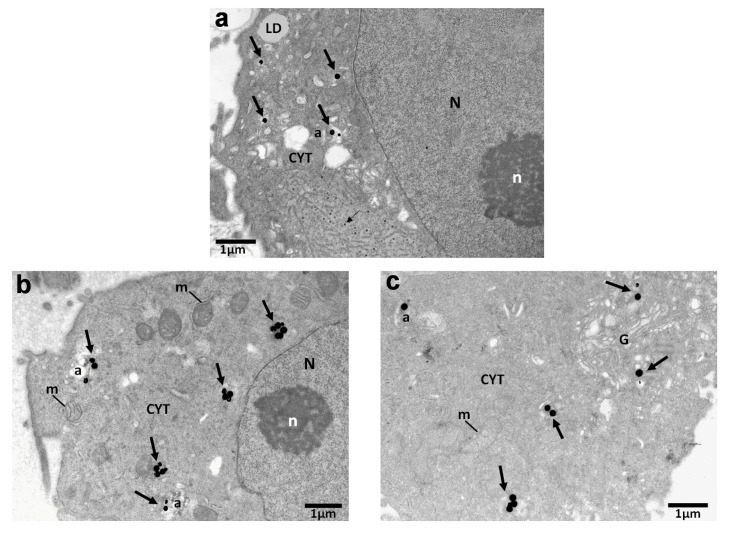
Electron micrographs of SiHa cells indicating the cellular uptake of 120 nm AuNPs (5 μg/mL) after 24 h. Images show the distribution and localization of AuNPs in different cells. Thick arrows point to the different areas of the cytoplasm, where nanoparticles were located. Nanoparticles were found inside vesicles, autophagosomes (**a**–**c**), or near the Golgi apparatus (**c**). Thin arrows a show HPV virus particles (virions) located in the cytoplasm. N: nucleus; n: nucleolus; CYT: cytoplasm; m: mitochondrion; a: autophagosome; G: Golgi apparatus; LD: lipid droplet. Scale bars: (**a**–**c**) 1 μm.

In order to investigate the cellular uptake of AuNPs after 24 h treatment, we applied TEM. Inside the SiHa cells, AuNPs were rarely located as single particles inside the cytoplasm. More commonly, nanoparticle agglomerates/aggregates were detected inside the cytoplasm and the majority of AuNPs were enclosed within membranous structures/vesicles or autophagosomes as shown in Figure 3a–c.

In some cases, vesicles and autophagosomes containing AuNPs as agglomerates were also found near the mitochondria and Golgi apparatus. Vesicles were identified as single-membrane structures containing only AuNPs, whereas autophagosomes were identified as double-membrane vesicles containing AuNPs, as well as degraded cellular material.

### 3.2. Cell Survival Curves

**Figure 4:**
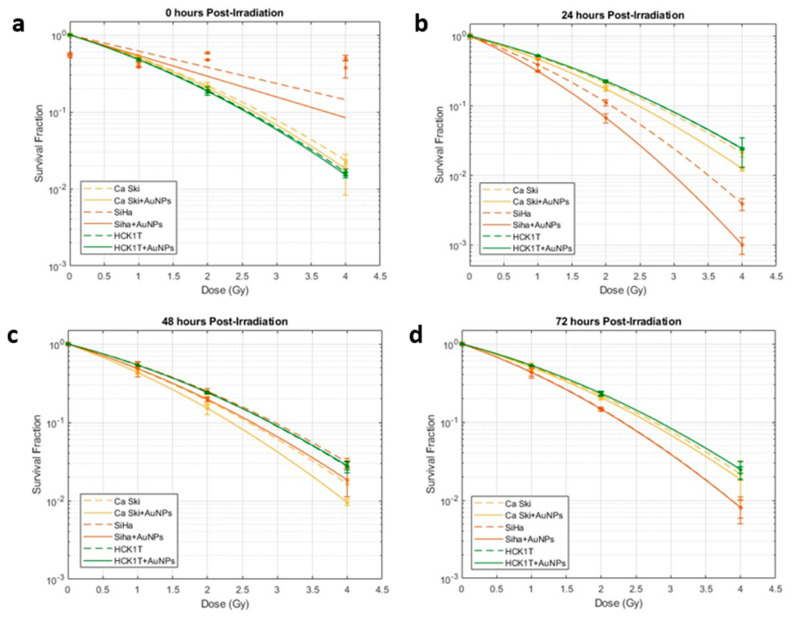
Radiation responses of cells and cells with 5 μg/mL of 120 nm gold nanoshells incubated for 24 h fitted with the linear quadratic model for different post−irradiation times and different deposited doses. Circles denote the mean survival at each dose point, and error bars indicate the standard deviation. Survival fractions over dose of Siha, Caski and HCK1T with AuNPs (solid lines) and without AuNPs (dashed lines) are depicted for (**a**) 0 h after irradiation. (**b**) 24 h after irradiation. (**c**) 48 h after irradiation. (**d**) 72 h after irradiation.

Τo adequately quantify ionizing radiation-induced cell death, we are required to access cells’ ability to form colonies post irradiation. The survival fraction of the colonies as a function of dose can be determined by using the clonogenic assay. Thereinafter, the enhanced radiation responses due to AuNPs incubation in the cells can also be evaluated. The radiation dose enhancement studies with AuNPs in vitro were carried out in SiHa and Caski cancer cell lines upon irradiation with clinically used LINAC 6 MV. The radiosensitizing results in the cancer cell lines were also compared with the AuNPs effects on the normal cervical epithelium cell line HCK1T upon irradiation. The radiation responses of cells and cells with 5 μg/mL of 120 nm gold nanoshells incubated for 24 h were observed for different post-irradiation times and different deposited doses as can be depicted in the cell survival curves of Figure 4a–d. 

In the context of the linear quadratic (LQ) model for radiobiological survival curves, the fit parameters *α* (alpha), *β* (beta), and *α/β* have been calculated from our model and are listed in Tables S1–S4 for the different post-irradiation times (available in the Supplementary Material). In the realm of radiosensitization research, understanding quadratic parameters holds profound significance. α represents the linear component of the LQ model while quantifying the sensitivity to low radiation doses. *β* represents the quadratic component of the LQ model and thus characterizes the response at higher doses. The ratio *a/b* is equivalent to elucidating the dominance of linear or quadratic components in the dose–response relationship. A higher *a/b* signifies a heightened sensitivity at low doses, while a lower ratio indicates a pronounced response at high doses.

### 3.3. Dose Enhancement Factor

**Figure 5:**
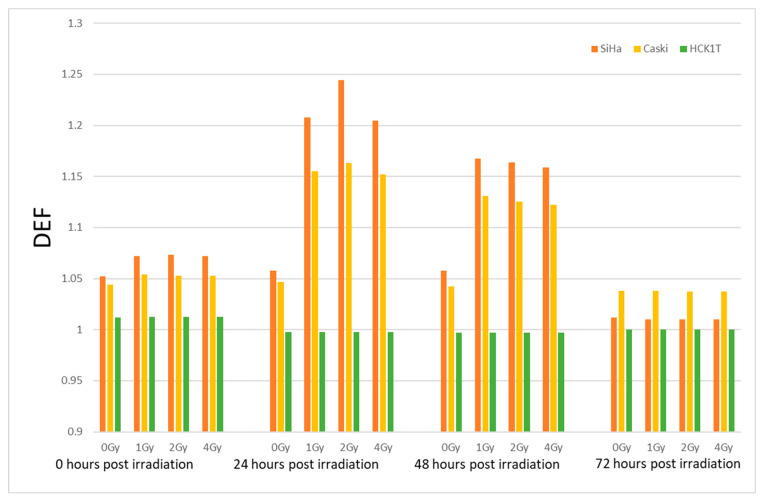
Dose Enhancement Factors for Siha, Caski, and normal cell line HCK1T after treatment with different doses and measured for different post-irradiation times.

The results for the clonogenic survival assays in response to the Dose Enhancement Factor for various cell lines, doses, and post- irradiation times are summarized in Figure 5.

By analysis of variance (Wilcoxon–Mann–Whitney test) for the 24 h post -irradiation time, a statistical significance was observed between Siha-Normal cells (*p* value = 0.022) and Siha-Caski cells (*p* value = 0.021). A statistical significance was also noted at the 2 Gy irradiation dose between Siha and Caski cells (*p* value = 0.021) for the different post-irradiation times. Comparing the DEF values for the post-irradiation times at 0 h and 24 h, Siha cells present a statistical difference (*p* value = 0.049), while the Caski cell response does not change statistically significantly with the post-irradiation time (*p* values > 0.1).

### 3.4. Apoptosis Measurements

**Figure 6:**
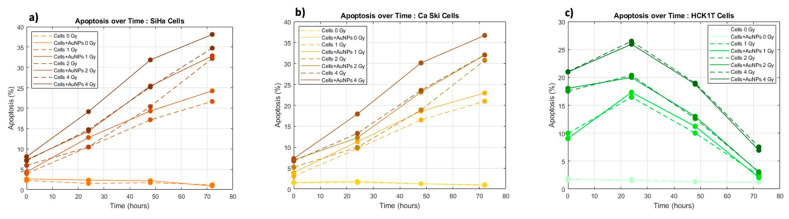
Depiction of apoptosis over time for cells and cells with AuNPs for the doses of 0 Gy, 1 Gy, 2 Gy, and 4 Gy for (**a**) Siha cervical cancer cell line. (**b**) Caski cervical cancer cell line. (**c**) HCK1T normal cervical cell line.

Simultaneously to the levels of cell death after treatment, the apoptosis levels of the cells have been measured in order to properly quantify and elucidate the programmed cell death due to ionizing radiation with and without AuNPs at different post- irradiation times and doses. The quantification of apoptosis over time for the two cervical cancer cell lines and the normal cervical cell line are depicted in Figure 6.

## 4. Discussion

The results presented in the radiobiological cell survival curves fitted with the linear quadratic (Figure 4), as well as the DEF calculations (Figure 5), and the apoptosis assessment (Figure 6) indicate variances and correlations that could be analyzed in three major domains: 1. the survival fractions with and without AuNPs and the possible dose enhancement, 2. the differences among cancer and normal cell lines while incubated with AuNPs and get treated in 6 MV LINAC, and 3. the post- irradiation time effects on the cell survival, cell cycle, and on the programmed apoptotic death with and without AuNPs.

### 4.1. Assessment of Distribution and Localization of AuNPs in Cells via TEM

The results indicated findings related to the radiosensitization of gold nanoparticles obtained through Transmission Electron Microscopy (TEM). TEM’s high magnification and resolution capabilities enabled the visualization of the distribution of AuNPs within specific cellular compartments and that way proved the cellular uptake of 120 nm AuNPs within the cells. According to research, the cellular uptake of AuNPs, as well as their location and their lifespan inside the cell, plays a major role in their radiosensitizing effects and physical mechanisms of action [25]. Even though AuNPs did not seem to enter the nucleus, they were often located near the perinuclear region (Figure 3a,b). Therefore, it is acceptable to say that the enhanced DNA damage related to the apoptotic levels we observe in Figure 6a,b can be attributed to AuNPs when their presence is near the perinuclear region or to the increase in Reactive Oxygen Species production after AuNP irradiation [26,27]. The results agree with previous findings regarding AuNP cellular uptake [28].

### 4.2. Assessment of the Cell Survival Curves

The fraction of cells surviving is plotted on a logarithmic scale over the dose, as can be depicted in Figure 4. In general, the survival fraction in LQ model can be assessed by observing the parameter of the slope. It is obvious that in terms of the slope, the levels of cell death increase with the increase in the dose (with a high dependency on the cell type and post-irradiation time). Throughout Figure 4, it can be observed that the surviving fraction remains a linear and then an exponential function of the dose (with high dependency on the cell type and post-irradiation time).

This can also be attested by the calculated fit parameters α (alpha), β (beta), and α/β (available at supplementary material). Both parameters are positive because they represent the degree of cell killing or damage caused by radiation. The positive α value means that cell killing increases linearly with the dose, while the positive *β* value means that cell killing increases quadratically with the dose. The higher values of *α* indicate a higher sensitivity to radiation in low doses, while the lower values of *β* represent the small contribution of the quadratic component in the low doses used. The calculated experimental values of α/β seem to agree with those of the literature where the ranges have been observed to be 0–10 Gy^−1^ [29] for various cancer types and more precisely 3.1–20.9 Gy^−1^ for normal cervical and cervical cancer cell lines [30].

### 4.3. Assessment of the Dose Enhancement

The 120 nm gold nanoshells’ enhanced radiation responses were evaluated through a clonogenic survival assay for the different doses, cell lines, and post-irradiation time. According to the depicted results in Figure 4, it is obvious that the levels of cell death are increasing not only with the increase in dose but also with the use of AuNPs. It is interesting to observe that the radiosensitization with AuNPs was abundant in all doses (at least for the cancer cell lines). This fact can also be attested in Figure 5, where the DEF is presented. The values ranged from 1.052 to 1.245 for the cancer cell lines, indicating a radiation enhancement contrary to the DEF values for the normal cell line which presented an absence of enhanced radiation responses in the range of 0.99–1.012 (for precise post- irradiation times). The acquired values of the DEF for our ex vivo experiments comply with the values of other research groups on the subject [31,32,33,34,35] with similar AuNP sizes used and for the same 6 MV energies.

An interesting observation of the DEF distribution against the dose can be seen in Figure 5, where it is observed that the relationship between the dose enhancement factor and the dose is not a simple linear one [36,37]. We are led to the observation that the DEF may increase with the dose of radiation up to a certain point, after which it reaches a plateau [38]. At low doses, there is a point where the DEF plateaus, meaning that increasing the radiation dose beyond a certain point does not significantly enhance the effect [39]. The plateau effect indicates that there is an optimal dose range for maximizing the DEF. Beyond this point, a further increase in the radiation dose might not significantly enhance the effect at least for the low dose range of 0–4 Gy. This observation can be attested by the literature and can be explained due to the multifactorial dependency of the DEF on the energy of radiation, nanoparticles characteristics, or the cell type specificity [40,41,42].

### 4.4. Assessment of Cancerous and Normal Cervical Cell Lines/AuNPs Response to Radiation

As can be observed in Figure 4, both of the cervical cancer cell lines, SiHa and Caski, seem to share a common pattern regarding their radiobiological behavior against the dose. The same pattern is also observed in Figure 5 regarding their DEFs. However, what can be distinguished is that the cell line of SiHa presents a higher slope of cell death increase with the increase in the dose in the LQ model compared to that of Caski. This consistency is obvious at the DEF levels in Figure 5 as well where the cell line of SiHa illustrates higher levels of the DEF compared to that of Caski for the same doses (and even post-irradiation times). The explanation of the phenomenon relies on the genomic identities of the cell lines. The Siha cell line was isolated from a 55-year-old, female patient and expresses the genes of p53+ and pRB+ [43]. The Caski cell line was isolated from a 40-year-old patient and does not express the p53 gene [44]. The literature has proposed that “the activation of p53 gene involve the transcriptional induction of redox-related genes with the formation of reactive oxygen species, leading to cell death by oxidative stress” [45,46]. Furthermore, it has been observed that p53-dependent apoptosis may be highly significant towards this direction. It is interesting to observe that in Figure 6, the apoptotic levels of SiHa and Caski still share a common pattern, and thus, the apoptotic levels of SiHa over time are higher.

The use of the normal human cervical keratinocytes cell line HCK1T and the results of Figure 4 can verify the concept that the radiation injury and the DNA damage starts immediately after irradiation for the normal cell line (Figure 4a). However, the DNA repair capacity of cells with damage from radiation therapy is in general higher in normal cells than in cancerous cells [47]. In other words, cancer cells are more susceptible to radiation than normal cells [47] over the progression of time because of the breakdown in cell cycle checkpoints and repair mechanisms. This increased radiosensitivity leads to the accumulation of irreparable DNA lesions and eventually higher levels of cell death for the cancerous cell lines compared to the normal one as can be depicted in Figure 4b–d.

Regarding the AuNPs incubation and treatment results, it is apparent that the cell survival rate decreases significantly for the cancer cell lines with AuNPs compared to the control cells, indicating severe cell damage to cancer cells, and enhanced localized dose deposition. On the other hand, very low to no radiation enhancement effect after the AuNPs use was observed on the healthy/noncancerous cells (Figure 4 and Figure 5), a fact that experimentally verifies the AuNPs selectively targeting on cancer cells while sparing healthy tissues [48]. This also attests to the enhanced permeability and retention (EPR) effect as well as the low systemic clearance of cancer cells compared to the low permeability of normal cells [49,50].

### 4.5. Assessment of the Post-Irradiation Time Effects

The significance of post-irradiation time for both cancer and normal cells on the immediate and delayed effects of ionizing radiation and accumulation of DNA damage, as well as the repair mechanisms, can be depicted in Figure 4. As can be observed, the maximum levels of cell death for the normal cell line are depicted at the 0–24 h post-irradiation, while after that, their ability to repair induced radiation damage and resume normal functions faster than the cancer cells is apparent. Contrary to that, the DNA damage in cancer cells that are preventing them from dividing and growing is maximizing at the 24 h post-irradiation, proving the delaying effects manifestations for cancer (Figure 2b). After 48 h, tumor cells repopulation occurs and the residual cells that have survived proliferate and re-establish the colony (Figure 4c,d). Figure 6 further shows the undergoing apoptosis of cancer cells, meaning that the programmed cell death will not occur initially after irradiation but will require at least 24 h.

Post-irradiation time seems to have an active role on the dose enhancement after the AuNP use, as can be seen in Figure 5. Even though the radiosensitization is apparent at every post-irradiation time, the maximum results are observed for 24 h with a steady decrease in the DEF for 48 and 72 h, respectively, when the AuNPs have been fully cleared from the cell environment.

### 4.6. Assessment of Apoptosis

The fundamental role of apoptosis as a controlled biological process towards programmed death after irradiation and its correlation with deposited dose, post-irradiation time, and AuNPs is depicted in Figure 6.

The role of apoptosis as a mechanism for cell death following ionizing radiation exposure can be depicted in all three cell lines, since the control group presents very low levels of apoptosis. As the deposited dose and as the post-irradiation time are increasing, the levels of lethal and sublethal damage are accumulating, leading to an increase in apoptotic levels. The interest presents the levels of apoptosis for the normal cell line, where contrary to the cancer cells, the apoptotic levels drop after 24 h since their cell cycle mechanisms allowed for faster repair of their DNA damages compared to the cancer cells.

In terms of AuNPs and their role in the induced apoptosis, it is observed that in the case of the cancer cell lines, the use of AuNPs significantly increases the levels of apoptotic cell death. The interest presents the apoptotic levels of 2 Gy with AuNPs which provides the same result as that of 4 Gy without AuNPs (Figure 6a,b). As has been previously discussed, the normal cell line indicates no or very low retention of AuNPs and thus no variations in the levels of apoptosis with gold nanoparticles.

To conclude, our findings can offer a promising avenue for the development of effective strategies as the cervical cancer’s asymptomatic nature in the early phases accentuates the critical need for a targeted therapeutic approach. Our results exhibit the selective uptake and localization of AuNPs in cancer cells, as well as the enhancement of radiation responses, as evidenced via TEM. The differential response between the cancer and normal cell lines, coupled with minimal radiation enhancement in normal cells, aligns with the imperative for precision in therapeutic interventions. The elucidation of post-irradiation time effects, demonstrating the temporal dynamics of cell death, DNA repair, and apoptosis, further refines our understanding of the therapeutic window for optimal treatment efficacy.

Despite the promising outcomes and potential implications of our research, it is essential to acknowledge certain limitations. One notable constraint lies in the variability in responses among diverse tumor subtypes. This necessitates further investigation on more cervical and normal cell lines for a more comprehensive understanding. Additionally, regarding the selective targeting and enhanced therapeutic effects of AuNPs, the complex interplay of various factors, including the tumor microenvironment dynamics and patient-specific characteristics, may influence treatment outcomes. The ex vivo nature of our experiments may not fully capture the complexities of an in vivo setting where the existence of the immune system and dynamic treatment responses will be present [26]. Addressing these limitations will be pivotal for advancing the translational potential of our research and ensuring its applicability across a broad spectrum of cervical cancer cases.

## 5. Conclusions

In this comprehensive study, the impact of gold nanoparticles (AuNPs) on cervical cancer radiotherapy was rigorously investigated. By meticulously analyzing cell survival curves, dose enhancement factors (DEFs), and apoptosis in both cancer and normal cervical cells, intricate radiobiological interactions were elucidated. The results demonstrate the enhancement of radiation responses in cancer cells when treated with AuNPs, leading to escalated cell death and apoptotic levels. Particularly noteworthy was the substantial enhancement effect observed in cancer cells, notably at the 24 h post-irradiation mark, emphasizing the critical role of timing in AuNPs’ efficiency. Importantly, AuNPs exhibited a discerning ability to target cancer cells while sparing normal cells, validating their potential as precise therapeutic agents. This further proves that the effectiveness of radiosensitization with gold nanoparticles depends on various factors, including the energy of the radiation used, the dose, the size, and concentration of the nanoparticles, the cell type, and the time the cell has to activate its repair mechanisms. The study’s findings illuminate the promising avenue of AuNPs as potent radiosensitizers in cervical cancer treatment, offering a targeted and effective approach to therapy. These insights not only deepen our understanding of the complex dynamics between AuNPs and cells but also pave the way for their optimized clinical application, potentially revolutionizing cervical cancer radiotherapy and advancing personalized cancer treatments.

## Data Availability

Data are available from the corresponding author upon reasonable request.

## Supplementary Materials

The following supporting information can be downloaded at: https://www.mdpi.com/article/10.3390/biom13121720/s1, Table S1: *α* (alpha), *β* (beta), and *α/β* parameters for 0 h post-irradiation; Table S2: *α* (alpha), *β* (beta), and *α/β* parameters for 24 h post-irradiation; Table S3: *α* (alpha), *β* (beta), and *α/β* parameters for 48 h post-irradiation; Table S4: *α* (alpha), *β* (beta), and *α/β* parameters for 72 h ost-irradiation.
