# Supplementary material for: Nanoparticle-Mediated Radiotherapy: Unraveling Dose Enhancement and Apoptotic Responses in Cancer and Normal Cell Lines
Source: Biomolecules. 2023 Nov 29;13(12):1720. doi: 10.3390/biom13121720 (PMC10742116; doi:10.3390/biom13121720)
Supplement: Supplementary file 1 [file biomolecules-13-01720-s001.zip › biomolecules-2721577-supplementary.pdf]

Supplementary Information

"Nanoparticle-Mediated Radiotherapy: Unraveling Dose Enhancement and Apoptotic Responses in Cancer and Normal Cell Lines "

Maria Anthi Kouri, Ellas Spyratou, Maria-Eleni Kalkou, Georgios Patatoukas, Evangelia Angelopoulou, Ioanna Tremi, Sophia Havaki, Vassilis G. Gorgoulis , Vassilis Kouloulias, Kalliopi Platoni, and Efstathios P. Eftstathopoulos*.

Table S1 α (alpha), β (beta) and α/ β parameters for 0 hours Post-Irradiation.

(**Gy**$^{-1}$)	**SiHa**		**Caski**		**HCK1T**	
	Cells	***Cells+AuNPs***	***Cells***	***Cells+AuNPs***	***Cells***	***Cells+AuNPs***
**α**	0.59	0.82	0.59	0.58	0.63	0.64
**β**	0.09	0.16	0.09	0.10	0.10	0.10
**α/β**	6.64	5.00	5.64	5.64	6.41	6.30

Table S2 α (alpha), β (beta) and α/ β parameters for 24 hours Post-Irradiation.

(Gy^{-1})	SiHa		Casky		HCK1T	
	**Cells**	**Cells+AuNPs**	**Cells**	**Cells+AuNPs**	**Cells**	**Cells+AuNPs**
**α**	0.81	0.98	0.59	0.66	0.56	0.56
**β**	0.15	0.19	0.10	0.11	0.09	0.09
**α/β**	5.59	5.29	6.06	5.64	6.05	6.05

Table S3 α (alpha), β (beta) and α/ β parameters for 48 hours Post-Irradiation.

(Gy^{-1})	SiHa		Casky		HCK1T	
	**Cells**	**Cells+AuNPs**	**Cells**	**Cells+AuNPs**	**Cells**	**Cells+AuNPs**
**α**	0.53	0.64	0.64	0.74	0.53	0.53
**β**	0.09	0.09	0.10	0.11	0.09	0.09
**α/β**	6.14	6.98	6.53	6.86	5.84	5.84

Table S4 α (alpha), β (beta) and α/ β parameters for 72 hours Post-Irradiation.

(Gy^{-1})	SiHa		Casky		HCK1T	
	**Cells**	**Cells+AuNPs**	**Cells**	**Cells+AuNPs**	**Cells**	**Cells+AuNPs**
**α**	0.71	0.72	0.56	0.59	0.54	0.54
**β**	0.12	0.12	0.10	0.10	0.10	0.10
**α/β**	5.70	5.93	5.67	5.68	5.58	5.58
